# Factors Determining Long-Term Antitumor Responses to Immune Checkpoint Blockade Therapy in Melanoma

**DOI:** 10.3389/fimmu.2021.810388

**Published:** 2022-01-11

**Authors:** Kimberly Loo, James W. Smithy, Michael A. Postow, Allison Betof Warner

**Affiliations:** ^1^ Department of Medicine, Memorial Sloan Kettering Cancer Center, New York, NY, United States; ^2^ Department of Internal Medicine, New York-Presbyterian Hospital and Weill Cornell Medicine, New York, NY, United States; ^3^ Department of Medicine, Weill Cornell Medical College, New York, NY, United States

**Keywords:** melanoma, immunotherapy, long-term response, biomarkers, survival

## Abstract

With the increasing promise of long-term survival with immune checkpoint blockade (ICB) therapies, particularly for patients with advanced melanoma, clinicians and investigators are driven to identify prognostic and predictive factors that may help to identify individuals who are likely to experience durable benefit. Several ICB combinations are being actively developed to expand the armamentarium of treatments for patients who may not achieve long-term responses to ICB single therapies alone. Thus, negative predictive markers are also of great interest. This review seeks to deepen our understanding of the mechanisms underlying the durability of ICB treatments. We will discuss the currently available long-term data from the ICB clinical trials and real-world studies describing the survivorship of ICB-treated melanoma patients. Additionally, we explore the current treatment outcomes in patients rechallenged with ICB and the patterns of ICB resistance based on sites of disease, namely, liver or CNS metastases. Lastly, we discuss the landscape in melanoma in the context of prognostic or predictive factors as markers of long-term response to ICB.

## Introduction

The success of immune checkpoint blockade (ICB) in the treatment of advanced melanoma reinvigorated clinicians and investigators seeking long-term treatment benefit for patients with cancer. ICB was rapidly adopted as frontline therapy for melanoma due to its potential for sustained clinical and survival benefits. The median overall survival (OS) for melanoma shifted from a dismal 9 months with dacarbazine in the pre-ICB era to a median OS of 6.5 years for patients treated with the combination nivolumab + ipilimumab on the CheckMate 067 trial (1). There is even the potential for long-term disease control after treatment discontinuation, a concept previously unrealized in the treatment of metastatic disease.

Despite the considerable promise of ICB, only about half of treated patients experience response, with many others experiencing primary or acquired resistance to ICB (2, 3). Additionally, toxicity from immune checkpoint blockade can be severe and even life-threatening, so identification of patients who are likely to benefit is of the utmost importance (4). Extensive studies are underway to uncover both prognostic and predictive biomarkers of long-term response, with the push to identify tumor-specific, tumor microenvironment, or T cell markers of long-term responders actively ongoing. Here, we describe the landscape of current clinical trials and real-world studies with long-term survival data following ICB treatment of advanced melanoma including rechallenge studies and highlight the prognostic and predictive biomarkers involved in the molecular determinants of tumor response and unique immune cell populations involved in extending the durability of ICB treatment response.

## Immune Checkpoint Blockade

Immune checkpoint blockade therapies aimed at harnessing adaptive immunity have driven a therapeutic revolution. Monoclonal antibodies against cytotoxic T lymphocyte antigen-4 (CTLA-4) and programmed death 1 (PD-1) capitalize on the inhibition of immune checkpoint pathways, creating several promising new avenues for new drugs in cancer therapy. CTLA-4 and PD-1 act as negative regulators of T cell immune function at different stages of the immune response (5).

CTLA-4 competitively binds CD80/CD86 with a higher affinity than CD28, which upon binding, dampens T cell activation and delivers inhibitory signals to the T cells (6) and has been shown to largely act in the lymph nodes at the initial priming stage of naive T cell activation by halting autoreactive T cells (7, 8). On the other hand, PD-1 is expressed on tumor cells, cells within the tumor microenvironment (TME), B cells, and natural killer (NK) cells (9, 10). PD-1 expression is induced upon T cell activation and inhibits the T cell receptor (TCR) “stop signal.” The activity of PD-1 inhibits kinases involved in T cell activation and affects the duration of T cell to antigen-presenting cell (APC) and T cell to target cell contact (11, 12). PD-1 has been described as another co-inhibitory receptor induced by T cell activation (13). PD-1 acts later in the immune response by regulating previously activated T cells predominantly in peripheral tissues during the T cell effector phase (14). Immune checkpoint blockade consequently utilizes the concept that tumor cells, typically recognized by T cells, have found ways to evade the immune system by utilizing peripheral tolerance (15, 16).

Ipilimumab, a monoclonal antibody against CTLA-4, was approved by the US Food and Drug Administration (FDA) in 2011. Pembrolizumab and nivolumab, both monoclonal antibodies against PD-1, gained FDA approval in 2014. Melanoma has especially benefited from the use of such immune checkpoint blockade agents. From several early clinical trials of these agents, ongoing studies demonstrating long-term survival are maturing. Prolonged overall survival and sustained clinical benefit even in patients who experienced stable disease to these ICB therapies have led to the current widespread use and favor of these agents in the first-line therapy setting in advanced melanoma.

Early promising results of anti-CTLA-4, ipilimumab, and anti-PD-1, pembrolizumab and nivolumab, demonstrated that ipilimumab compared favorably to the current standard melanoma therapies of the time including gp100 peptide vaccine and improved overall survival (OS) (17). Anti-PD-1 antibodies quickly followed suit to add to the repertoire of ICB agents. Initial clinical trials demonstrated response rates of 20%–40% in melanomas treated with anti-PD-1 agents with prolonged stabilization of disease and lower severity and frequencies of grade 3–4 adverse events compared with chemotherapy and ipilimumab (18–21).

Notably, the KEYNOTE-001 study of pembrolizumab demonstrated a robust objective response rate (ORR) in ipilimumab refractory patients (22). The KEYNOTE-002 study demonstrated improved progression-free survival (PFS) in patients who received pembrolizumab compared with those who received investigator choice chemotherapy (23). The KEYNOTE-006 study demonstrated superior OS and PFS in patients treated with pembrolizumab compared with ipilimumab in ICB treatment-naive patients with improved grade 3–4 adverse events (2, 24). The CheckMate 037 trial demonstrated improved ORR in patients treated with nivolumab compared with investigator choice chemotherapy in patients who had previously progressed on ipilimumab or BRAF inhibitors of the BRAF mutant (25). The CheckMate 066 study demonstrated improved median PFS, ORR, and 1-year OS rate in previously untreated BRAF wild-type patients treated with nivolumab compared with dacarbazine (26).

The combination of immunotherapies then followed given the clinical success experienced by ICB monotherapies. Nivolumab in combination with ipilimumab (nivo + ipi) has been associated with response rates of up to 58% and 22% of complete response to treatment (27–29). The CheckMate 067 trial compared the nivo + ipi combination to ipilimumab monotherapy. These results demonstrated significantly improved ORR, PFS, and OS in the nivo + ipi combination group compared with ipilimumab (27). CheckMate 064, a randomized phase II study, compared the sequential treatment of ipilimumab and anti-PD-1 rather than in combination as what the CheckMate 067 trial had conducted. Two arms included a nivolumab induction followed by ipilimumab then nivolumab maintenance arm vs. an ipilimumab induction then nivolumab with nivolumab maintenance. Efficacy outcomes were superior in patients treated with nivolumab frontline therapy compared with initiation with ipilimumab, with statistically similar toxicity rates. Median OS was not reached (30). Substantially improved objective response, PFS, and OS irrespective of BRAF status have propelled the nivo + ipi combination as a standard of care in melanoma, despite increases in grade 3–4 adverse events in patients treated with the combination (3, 31, 32).

## Long-Term Outcomes to ICB

Across clinical trials and now with years of clinical experience, the early promise of durable melanoma control with ICB is coming to fruition. We are now seeing survival data for 7 years and beyond post-ICB treatment with the tail of survival curves maturing to provide the promise of durable disease control and long-term treatment outcomes in melanoma ICB-treated patients.

KEYNOTE-001 evaluated 655 patients with advanced melanoma treated with pembrolizumab. With a median follow-up of 55 months, the estimated 5-year OS was 34% in all patients in the study and 41% in treatment-naive patients. The median duration of response was not yet reached at the 5-year timepoint. Seventy-three percent of responses in the entire cohort were ongoing, and 82% of treatment-naive responses were ongoing. The longest response was ongoing at 66 months at the time of data cutoff. Four patients who initially had a complete response (CR) and discontinued therapy ultimately experienced disease progression and were retreated with a second course of pembrolizumab. Two of the four patients had disease response (33).

Similarly, the 5-year *post-hoc* analysis results of the KEYNOTE-006 trial of ipilimumab-naive patients treated with pembrolizumab or ipilimumab were reported (34). Participants with stable disease (SD) or better after receiving at least 24 months of treatment or CR after at least 6 months of pembrolizumab stopped the therapy per protocol. With a median follow-up in survivors of 57.7 months, the median OS was 32.7 months (95% CI: 24.5–41.6) in the pembrolizumab-treated group versus 15.9 months (13.3–22.0) in the ipilimumab-treated group (*p* = 0.00049). This trial not only confirmed the superiority of PD-1 blockade over ipilimumab, but it also showed that the long-term follow-up data further support the durability of ICB responses.

The exploratory 7-year follow-up data of KEYNOTE-006 (KEYNOTE-587) have recently been presented by Robert et al. at the Society of Melanoma Research 2021 Congress (35). Following the conclusion of KEYNOTE-006, 210 eligible patients transitioned to KEYNOTE-587 for extended follow-up (158 received pembrolizumab, 52 received ipilimumab). The median OS was 32.7 months for pembrolizumab-treated patients versus 15.9 months for ipilimumab-treated patients (HR = 0.70; 95% CI: 0.58–0.83). The 7-year OS rates were 37.8% in pembrolizumab- versus 25.3% in ipilimumab-treated patients. Pembrolizumab was associated with improved clinical outcomes regardless of prior BRAF inhibitor therapy, large tumor burden, elevated LDH, or prior brain metastases.

The CheckMate 067 trial compared nivolumab + ipilimumab with ipilimumab alone. The 6.5-year follow-up data from this study were recently presented, confirming previously reported sustained efficacy. With a minimum follow-up of 6.5 years, the median OS was 72.1 months (38.2–NR), 36.9 months (28.2–NR), and 19.9 months (16.8–24.6) in the nivo + ipi combo, nivolumab monotherapy, and ipilimumab monotherapy arms, respectively. Importantly, the median treatment-free interval (excluding patients who discontinued follow-up prior to subsequent systemic therapy) was 27.6 months in the combination immunotherapy arm, reinforcing the durability of benefit even after treatment discontinuation (1).

Long-term recurrence-free survival results are also beginning to mature for adjuvant ICB for patients with high-risk resected stage III/IV melanoma. The efficacy of adjuvant therapy addresses a slightly different clinical scenario—that of micrometastatic disease. Thus, durable benefit after adjuvant therapy is suggestive of long-term efficacy against microscopic disease as well as detectable metastases. The CheckMate 238 trial demonstrated 4-year results from adjuvant nivolumab versus ipilimumab in resected stage IIIB–C and stage IV melanoma. This multicenter, double blind, randomized controlled phase III trial demonstrated sustained recurrence-free survival benefit in patients treated with nivolumab compared with ipilimumab. Median follow-up was 51.1 months with adjuvant nivolumab and 50.9 months with adjuvant ipilimumab. The 4-year recurrence-free survival was 51.7% (95% CI: 46.8–56.3) in the nivolumab group and 41.2% (36.4–45.9) in the ipilimumab group (*p* = 0.0003). The 4-year OS was 77.9% with nivolumab and 76.6% with ipilimumab (*p* = 0.31) (36). This study demonstrates the sustained long-term benefit of adjuvant nivolumab compared with ipilimumab in patients with high-risk resected melanoma, especially also considering a more favorable toxicity profile in anti-PD-1-treated patients. Similarly, KEYNOTE-054 demonstrated improved 3.5-year distant metastasis-free survival with pembrolizumab versus placebo at a median of 42.3 months of follow-up (37). The efficacy of nivolumab and pembrolizumab is therefore expected to be similar (**Table 1**).

**Table 1:** Melanoma clinical trials with long-term survival results.

Trial	Treatment arms	Median follow-up	Median PFS/recurrence-free survival/intracranial PFS (95% CI)	Median OS/distant metastasis-free survival (95% CI)
KEYNOTE-001 (33)	Pembrolizumab monotherapy	55 months	Median PFS was 8.3 months (95% CI: 5.8–11.1) in all patients and 16.9 months (95% CI: 9.3–35.5) in treatment-naive patients	Median OS was 23.8 months (95% CI: 20.2–30.4) in all patients and 38.6 months (95% CI: 27.2–not reached) in treatment-naive patients
(NCT01295827)	Total melanoma patients (*n* = 655); treatment naive (*n* = 151) or previously untreated (*n* = 496)		5-year PFS rates were 21% in all patients, 29% in treatment-naive patients	5-year OS rates: 34% in all patients, 41% in treatment-naive patients
KEYNOTE-006 (34)	Pembrolizumab monotherapy or ipilimumab monotherapy	57.7 months	Median PFS was 8.4 months (95% CI: 6.6–11.3) in the combined pembrolizumab groups versus 3.4 months (95% CI: 2.9–4.2) in the ipilimumab group (HR 0.57, 95% CI: 0.48–0.67, *p* < 0.0001)	Median OS was 32.7 months (95% CI: 24.5–41.6) in the combined pembrolizumab groups and 15.9 months (95% CI: 13.3–22.0) in the ipilimumab group (HR 0.73, 95% CI: 0.61–0.88, *p* = 0.00049)
(NCT01866319)	Total (*n* = 834); pembrolizumab 10 mg/kg every 2 weeks (*n* = 279), 10 mg/kg every 3 weeks (*n* = 277), or ipilimumab 3 mg/kg every 3 weeks (*n* = 278)			
KEYNOTE-587 (35)	Pembrolizumab monotherapy or ipilimumab monotherapy	7-year follow-up data	Not reported	Median OS was 32.7 months for pembrolizumab-treated patients versus 15.9 months for ipilimumab-treated patients (HR 0.70, 95% CI: 0.58–0.83)
(NCT03486873)	Extended follow-up after conclusion of KEYNOTE-006 (*n* = 210); pembrolizumab (*n* = 158) or ipilimumab (*n* = 52)			7-year OS rates: 37.8% for pembrolizumab and 25.3% for ipilimumab
CheckMate 067 (1)	Nivo + ipi or nivolumab monotherapy or ipilimumab monotherapy	Minimum follow-up of 6.5 years	Median PFS: 11.5 months (95% CI: 8.7–19.3) nivo + ipi, 6.9 months (5.1–10.2) nivolumab, 2.9 months (2.8–3.2) ipilimumab	Median OS: 72.1 months (38.2–NR) nivo + ipi, 36.9 months (28.2–NR) nivo, and 19.9 months (16.8–24.6) ipi
(NCT01844505)	Nivo + ipi (*n* = 314), nivolumab only (*n* = 316), or ipilimumab only (*n* = 315)		6.5-year PFS rates: 34% (95% CI: 29%–40%) nivo + ipi, 29% (95% CI: 23%–34%) nivolumab, 7% (95% CI: 4%–11%) ipilimumab	6.5-year OS rates: 49% (95% CI: 44%–55%) nivo + ipi, 42% (95% CI: 37%–42%) nivolumab, 23% (95% CI: 19%–28%) ipilimumab
CheckMate 238 (36)	Adjuvant nivolumab monotherapy or ipilimumab monotherapy	51.1 months in adjuvant nivolumab	4-year recurrence-free survival was 51.7% (95% CI: 46.8–56.3) in the nivolumab group and 41.2% (36.4–45.9) in the ipilimumab group (*p* = 0.0003)	4-year OS was 77.9% in the nivolumab-only group and 76.6% in the ipilimumab-only group (*p* = 0.31)
(NCT02388906)	Total (*n* = 453); adjuvant nivolumab only (*n* = 453) or adjuvant ipilimumab only (*n* = 453)	50.9 months in adjuvant ipilimumab		
KEYNOTE-054 (37)	Adjuvant pembrolizumab monotherapy or placebo	42.3 months	3.5-year recurrence-free survival was 59.8% (95% CI: 55.3%–64.1%) in the pembrolizumab group and 41.4% (95% CI: 37.0%–45.8%) in the placebo group (HR 0.59, 95% CI: 0.49–0.70)	3.5-year distant metastasis-free survival was 65.3% (95% CI: 60.9%–69.5%) in the pembrolizumab group and 49.4% (95% CI: 44.8%–53.8%) in the placebo group (HR 0.60, 95% CI: 0.49–0.73, *p* < 0.0001)
(NCT02362594)	Total (*n* = 1,019); adjuvant pembrolizumab (*n* = 514) or adjuvant placebo (*n* = 505)			
CheckMate 204 (93)	Nivo + ipi with active melanoma brain metastases	34 months	36-month intracranial PFS rate (icPFS): 54% (95% CI: 43%–64%) cohort A, icPFS 19% (95% CI: 5%–40%) cohort B	OS rate 72% (95% CI: 43%–64%) cohort A, 37% (95% CI: 14%–60%) cohort B
(NCT02320058)	Total (*n* = 119); cohort A: asymptomatic (*n* = 101) or cohort B: symptomatic and/or steroid requiring (*n* = 18)			
ABC trial (91)	Nivo + ipi or nivolumab monotherapy with active melanoma brain metastases (mets)	54 months	5-year icPFS: 46% cohort A, 15% cohort B, 6% cohort C	5-year OS rates: 51% cohort A, 34% cohort B, 13% cohort C
(NCT02374242)	Total (*n* = 76); asymptomatic brain mets with no prior local brain therapy			
	Cohort A: nivo + ipi (*n* = 35), cohort B: nivolumab only (*n* = 25), or cohort C: brain mets, previous local therapy with neuro symptoms and/or with leptomeningeal disease, nivolumab only (*n* = 16)			

It is important to note that most of the long-term data discussed to date have been from clinical trials. Given the differences in clinical trial populations and real-world outcomes, data are needed from patients who received standard of care. In a large single-institution retrospective study of patients treated with anti-PD-1, those who discontinued therapy and had at least 3 months of follow-up (*n* = 396) were evaluated for durability of long-term response as well as retreatment outcomes following anti-PD-1 disease progression. Median OS was 39 months (31.7–47.2 months) and 5-year OS was 40.8% (33.7–47.8%). One hundred and two (25.8%) patients experienced CR to anti-PD-1. Median follow-up was 21.1 months from the time of CR in patients who did not relapse. This study demonstrated that most CRs to anti-PD-1 were durable, yet the probability of treatment failure at 3 years was 27%. Additionally, of the patients who achieved CR to a single-agent anti-PD-1, 23 of these CR patients later experienced progressive disease (38).

In terms of the immune-related adverse event (irAE) profile experienced during ICB treatment that correlated with long-term response, the development of irAE hypothyroidism and vitiligo within 6 months of treatment was associated with long-term OS (median 43.6 vs. 13.1 months in those without irAEs, *p* = 0.008) (39). Vitiligo has been observed as an irAE linked with durable response and lower risk of progression or death in melanoma, likely due to the shared antigen between benign melanocytes and melanoma cells (40). Similarly, those who have experienced the irAE of thyroid dysfunction had significantly longer PFS and OS in another study, with prolonged survival long after disease progression (39, 41).

As the ICB arsenal continues to improve long-term survival outcomes across melanoma, the discussion of characteristics of survivorship, namely, chronic immune toxicities, functional status, and health outcomes, is actively being addressed in survivorship clinics. In patients treated with ipilimumab for metastatic disease or with adjuvant therapy with overall survival of >2 years, Johnson et al. describe the overall excellent functional outcome and toxicities experienced among long-term survivors. While chronic endocrine dysfunction and occasional neurologic toxicities (associated with whole brain radiation) were seen in a small number of surviving patients, gastrointestinal and dermatologic adverse events were the most frequent, though transient compared with those patients with hypophysitis who required ongoing corticosteroid treatment. Furthermore, surviving patients generally had excellent Eastern Cooperative Oncology Group (ECOG) performance statuses (ECOG 0–1), which is reassuring of life years following ipilimumab treatment (42).

Survivorship and health-related quality of life outcomes in patients experiencing durable responses to anti-PD-1/PD-L1 are also described by Patrinely et al. Among survivors greater than 2 years out from anti-PD-1/PD-L1 treatment for melanoma, renal cell carcinoma, or non-small cell carcinoma, ECOG performance status was 0 or 1 at last follow-up. Chronic irAEs which persisted beyond 12 weeks after anti-PD-1 discontinuation seen at follow-up included hypothyroidism, arthritis, adrenal insufficiently, and neuropathy, though no clear chronic adverse cardiometabolic events were observed (43). Characterization of chronic irAEs of patients treated with anti-PD-1 in the adjuvant setting is described by Patrinely et al. Chronic irAEs were common and persisted with prolonged follow-up, though most were mild low grade 1 or 2. Among patients who received adjuvant anti-PD-1 treatment, endocrinopathies, arthritis, xerostomia, and neurotoxicities were the most common. Additionally, irAEs affecting visceral organs such as the liver, colon, kidneys, and lungs were much less common to become chronic irAEs (44). Collectively, the favorable health-related outcomes among long-term survivors to ICB treatments are overwhelmingly reassuring as patients begin to transition to survivorship clinics for monitoring long after their ICB treatments.

In real-world studies examining patients who electively discontinued anti-PD-1 in the absence of disease progression or treatment limiting toxicity, the duration of anti-PD-1 treatment was shorter compared with the reported treatment course of patients treated on clinical trials (45). In a study of 185 patients treated across multiple centers across Europe and Australia, of the patients who electively discontinued anti-PD-1, those who experienced a CR (63%) and were treated for more than 6 months exhibited a lower risk of relapse after treatment discontinuation. Patients who achieved a PR (24%) or SD (9%) had a higher risk of disease progression after therapy discontinuation (NCT02673970) (46). Further studies to determine the optimal duration of treatment in patients who achieve PR or SD are needed.

In a separate real-world observational cohort study, patients who made a joint decision with their provider to electively discontinue anti-PD-1 therapy at 1 year (>6 and <18 months) were reviewed. Here, the majority of patients with metastatic melanoma following 1 year of anti-PD-1 treatment remained without progression in the long-term follow-up evaluation, with a low risk of disease progression even in patients with residual disease on imaging. Median follow-up in this cohort study was 20.5 months from anti-PD-1 treatment discontinuation with 75% of patients remaining without disease progression, while 25% had disease progression, with a median PFS of 3.9 months (range 0.7–30.9 months) (47). Given this, elective discontinuation of anti-PD-1 therapy may still achieve favorable long-term outcomes while also reducing the immunotherapy-related toxicities and financial burdens associated with prolonged anti-PD-1 treatment.

In a single cohort study examining patients with advanced melanoma treated with anti-PD-1 monotherapy of nivo + ipi, multivariate analysis revealed that patients with a non-CR to treatment as best overall response (BOR) and in cases where immunotherapy was given in the advanced line (where previous lines of treatment included ipilimumab monotherapy, targeted therapy, prior pembrolizumab, or nivo + ipi) should be treated for longer periods of time, with elective discontinuation discouraged prior to the 18-month timepoint (48). Finally, in another study examining CR in patients following anti-PD-1 treatments, 102 patients stopped treatment after a CR after a median duration of 9.4 months. Here, with a median follow-up of 21.1 months from the time of CR, the probability of being alive and not requiring additional treatment was 72.1% with an estimated 3-year OS from the time of CR of 82.7% (95% CI: 67.9%–91.1%) (38).

A prospective, multicenter single-arm interventional study in the Netherlands, the Safe Stop trial, examined patients with melanoma and a confirmed CR or PR to be included in this study examining early discontinuation of first-line monotherapy with the anti-PD-1 therapies pembrolizumab or nivolumab. The primary objective was to examine the rate of response 24 months following anti-PD-1 treatment discontinuation, with secondary objectives examining BOR and duration of response with need and outcomes of anti-PD-1 rechallenge and associated serious adverse events and health-related quality of life measures (49).

Beyond the impressive nature of the 5-plus year landmarked OS rates, the feasibility of determining functional cure rates in melanoma patients treated with ICB is actively emerging. A pooled analysis from several phase II and III studies of ipilimumab-treated patients demonstrated a plateau of survival curves at around year 3. The median OS in this cohort of 254 patients was 11.4 months (95% CI: 10.7–12.1). Follow-up in this cohort was reported for up to 10 years following ipilimumab initiation (50). With a plateau and flattening of the tail of the ipilimumab overall survival curves, thoughts surrounding functional cure rates with patients treated with melanoma are being discussed with much excitement. With the newly maturing ipilimumab data, ongoing analysis of patients treated with PD-1 and examination of the potential plateau curve are ongoing. For the first time, statistical evaluation with cure models may be possible.

We recently examined a subset of melanoma patients treated with ICB regimens who survived at least 5 years (*n* = 151). The median duration of response among survivors (*n* = 138) was 93 months. From the 5-year post-initial ICB timepoint, 85% of patients survived an additional 5 years (95% CI: 73%–92%). Among patients who made it to the 5-year post-ICB timepoint without treatment failure (*n* = 72), the probability of remaining treatment failure free at 7 years was 92% (86%–99%). Of the 151 patients, none ultimately died of melanoma (51). Given this, patients who survived at least 5 years following initial ICB demonstrated excellent sustained survival and treatment failure free years, a finding which is greatly reassuring to clinicians and patients.

## ICB Treatment Rechallenge

Despite the ability of ICB therapies to provide sustainable antitumor responses in a subset of patients, up to 25%–30% of patients experience recurrence of their melanoma within 1 year of treatment, and more than 50% eventually progress following ICB therapy (20, 52, 53). Approaches to rechallenge or subsequent therapies are being explored for those patients who eventually progress following ICB. Rechallenge regimens utilize repeated treatments with the same therapeutic class of drug following disease progression in patients who experienced previous clinical benefit with prior treatment for unresectable or metastatic disease (54, 54). This is typically considered because there are few effective treatment options for melanoma after progression on ICB. Rechallenge may be considered if initial treatment was discontinued for toxicity or if disease progression necessitates another line of therapy.

Retreatment with monotherapy ipilimumab has resulted in tumor response rates of 12% to 23% (55–57). In patients treated with single-agent anti-PD-1, retreatment with anti-PD-1 or nivo + ipi has led to objective responses in only 15% to 25% of retreated patients. The study by Betof Warner et al. demonstrates that responses to retreatment were infrequent among patients who experienced disease progression on anti-PD-1 and were subsequently treated with either anti-PD-1 or nivo + ipi. Seventy-eight (19.7%) patients who discontinued anti-PD-1 for any reason were subsequently treated with ICB; 45.6% of patients received PD-1 monotherapy and 56.4% received nivo + ipi. A total of 14.7% exhibited a response to PD-1 monotherapy and two patients achieved a CR. Twenty-five percent of patients exhibited a response to nivo + ipi and three patients achieved a CR (38).

Chapman et al. recently reported that in the retreatment of patients using the combination of nivo + ipi, the BOR and time-to-treatment failure (TTF) rates were markedly less favorable following nivo + ipi reinduction compared with the initial treatment course. Rechallenge of 26 patients who received the nivo + ipi combination demonstrated a BOR rate (complete response and partial response) of 74% following the first course of combination treatment versus 23% after reinduction. TTF was also shorter for reinduction compared with the first course in 85% of patients (58). Hepner et al. described the reinduction of 47 patients with ipilimumab (alone or in combination with anti-PD-1) after progressing on nivo + ipi therapy. Modest clinical activity was seen in this cohort despite the recurrence of immune-related adverse events occurring during the reinduction of 40% of this cohort. The response rate to reinduction was 26% at 5 months and the disease control rate was 45%. The median follow-up time of this study was reported as 16 months (95% CI: 10–25 months). The median PFS among responders to reinduction was 14 months (95% CI: 13–NR months). The median OS from reinduction for the entire cohort was 17 months (95% CI: 12–NR months) (59). Finally, Olson et al. have shown that in patients who progressed on anti-PD-1/PD-L1 therapy, retreatment with ipilimumab plus pembrolizumab had a 29% response rate with a median PFS of 5 months, median OS of 24.7 months, and median duration of response 16.6 months (60).

In a review of current studies of ICB treatment rechallenge, the mean disease control rate (DCR) and mean ORR were examined in several rechallenge groups. In rechallenge with anti-PD-1 following disease progression on PD-1, the mean DCR was 45.8% with a mean ORR of 15.5%. The mean DCR of 40.6% and the mean ORR of 20% were noted in patients rechallenged with nivo + ipi following disease progression on anti-PD-1. Rechallenge with anti-CTLA-4 following progression on anti-CTLA-4 demonstrated a mean DCR of 50.9% and a mean ORR of 20.4% (61).

Given the lower objective response rates, shorter time to treatment failure, and increased toxicities associated with ICB retreatment, the risks and benefits of ICB retreatment currently mirror the risk/benefit profile of several chemotherapies used for other malignancies. To obtain more robust prolonged survival on initial ICB regimens, the need to understand the mechanisms of resistance to ICB is heightened as these underlying patterns of resistance may be contributing to the decreased efficacy of retreatment courses. Identifying the cell populations associated with response or resistance to ICB is imperative to guide the development of agents that may provide long-term survival benefit similar to that of ICBs. Additionally, identifying new agents that may utilize different mechanisms of action, either independently or in synergy with ICBs, is imperative to treat those patients who may not respond to ICB and would not benefit from retreatment.

## Baseline Peripheral Blood Laboratory Factors Associated With ICB Outcome

Improved survival outcomes to ICB have been observed in patients with favorable prognostic factors. Prognostic factors include measures that are associated with clinical outcomes irrespective of therapy. Conversely, predictive markers include those factors associated with response or lack or response to therapeutic intervention (62, 63). The differentiation between prognostic versus predictive markers of long-term response is an important distinction given the varying relationships between prognostic or predictive biomarkers and clinical outcomes. Here, prognostic markers are thought to be a measure of the natural history of the disease where factors are measured prior to therapy such as lactate dehydrogenase (LDH), baseline neutrophil to lymphocyte ratios (NLR), or tumor burden. While low baseline tumor burden has been associated with favorable prognosis in melanoma, tumor burden can be measured and reported in several ways [i.e., tumor volume, tumor diameter (largest or combined), or number of metastases] and has not been incorporated into the American Joint Committee on Cancer (AJCC) staging guidelines (64, 65). Here, we describe baseline peripheral blood laboratory prognostic markers associated with long-term ICB response.

Baseline LDH has been a prognostic factor that has been widely utilized in melanoma. LDH has been previously shown to be an independent predictor of overall survival in melanoma and has been incorporated into the AJCC staging classification (66). Increased glycolysis uptake in cancer cells with accelerated metabolism generates elevated levels of LDH as a by-product, which has served as a proxy to assess melanoma tumor burden (67). However, LDH does not always correlate with tumor burden, and tumor size remains an independent prognostic marker (68). The AJCC staging classification has incorporated LDH as a prognostic marker, and studies have described elevated pretreatment LDH with poor OS outcomes in patients treated with ipilimumab and pembrolizumab (66, 69, 70). More recently, several studies have shown that LDH greater than twice the upper limit of normal when measured at baseline prior to ICB therapy correlates with poor response to ipilimumab and anti-PD-1 therapy (71, 72).

In a retrospective study of patients with advanced melanoma who received ICB monotherapy, elevated LDH, the extent of disease, and lymphopenia (<1,000 cells/μl) within 3 months of ICB start were associated with poorer OS and PFS outcomes. CheckMate 067 reported a difference in clinical benefit which was especially robust in patients with BRAF mutation-positive tumors, higher LDH levels (>2× the upper limit of normal, ULN), and those with M1c stage, although the frequency and severity of irAEs were much higher in the nivo + ipi combination regimen compared with nivolumab or ipilimumab monotherapy. Here, there was also a trend for improved survival in patients who received the combination and those with normal LDH or normal LDH with fewer sites of disease (27).

Additional prognostic markers of long-term response have been noted in baseline lymphocyte, neutrophil, and eosinophil levels in the peripheral blood. The absence of lymphocytes given lymphopenia has also been noted as a poor indicator of ICB response. Lymphocytes are crucial mediators in the mechanism of immune checkpoint inhibitors, with circulating lymphocytes often infiltrating tumors. The depletion of such immune cells may be a contributing factor to suboptimal ICB treatment response. Studies have shown that with ipilimumab, increases in absolute lymphocyte count 2–8 weeks after treatment as well as CD4^+^ and CD8^+^ T cells at 8–14 weeks were associated with improved OS and clinical response (partial or complete response to therapy) (73).

Eosinophils have been shown to contribute to tumor surveillance and to play an important role in tumor rejection in animal models (74, 75). In studies of ipilimumab-treated patients, high relative eosinophil count (REC) at baseline correlated with improved OS, and increases in REC levels early in treatment were associated with improved clinical response (76). REC ≥1.5% and relative lymphocyte count ≥17.5% were associated with favorable OS in patients treated with pembrolizumab. This was confirmed in a validation cohort and strongly associated with prognosis (77, 78). Additionally, eosinophils also correlate with irAEs. Studies have demonstrated that patients who develop eosinophilia on ICB treatment had significantly longer survival (79).

Elevated baseline NLR and increased NLR early in anti-PD-1 monotherapy treatment in patients with melanoma may serve as additional predictive markers for TTF and OS. A baseline NLR >5 was associated with shorter OS and TTF. An increase in NLR by more than 30% after two treatment cycles was associated with worse OS (median 47 vs. 13.5 months, *p* < 0.001) and trended toward a shorter TTF (12.8 vs. 5.9 months, *p* = 0.05) (80). Several other studies have similarly reported that in stage IV melanoma patients treated with nivolumab or ipilimumab, elevated baseline NLR >5 had significantly worse OS and performance status compared to patients with baseline NLR <5 (81, 82). High platelet to lymphocyte ratios (PLR) have also been shown to correlate with shorter OS but not PFS in melanoma patients. At a PLR cutoff of <120, subgroup analysis of nine studies indicated that PLR served as a significant prognostic indicator in both OS and PFS in patients with melanoma (83).

## ICB Resistance by Disease Sites and Liver and Brain Microenvironments

Sites of distant metastases have been studied both preclinically and in the clinical setting regarding the increase in mortality, resistance to ICB treatment, and immune tolerance mechanisms that may contribute to overall treatment outcomes. In particular, liver metastases and brain metastases have proven to be challenging in immune-oncology-based therapies. In patients with melanoma, the presence of liver metastases prior to ICB start negatively correlates with immunotherapy efficacy (84, 85). Independent of tumor burden, age, gender, and prior therapies, the presence of melanoma liver metastases is associated with worse outcomes in terms of inferior OS and PFS rates compared to those without liver metastases or those with only lung metastases. Patients with liver metastases were also more likely to have increases in systemic tumor burden compared with those without liver metastases. In the CheckMate 067 trial, participants with liver metastases treated with nivo + ipi had a median OS of 28.2 months compared with 72.1 months in the cohort overall (1).

In addition to the idea that the liver is a tolerogenic organ, many hypothesize that the presence of liver metastases may alter systemic antitumor activity. Studies have reported phenotypic changes to effector tumor-infiltrating lymphocytes in distant biopsy sites in patients with liver metastases (86). Of the patients with liver metastases, the fraction of partially exhausted cytotoxic T cells (peCTLs) was reduced. Moreover, in these patients with low levels of partially exhausted cytotoxic T lymphocytes, the combination nivo + ipi was associated with significantly higher objective response rates when compared with anti-PD-1 monotherapy. Furthermore, specific T cell populations have been detected in relative abundance in patients who have achieved clinical response to anti-PD-1 therapy. These partially exhausted tumor-infiltrating CD8^+^ T cells strongly correlated with response and PFS to anti-PD-1 therapy (87). Various populations of CD8^+^ T cells and their relative location and activation status in patients with liver metastases have been an active area of interest to decipher the underlying mechanism of liver metastases and response to ICB therapy.

In preclinical mouse models, liver metastases were shown to create a systemic immune desert and modulate the immune function in patients with solid tumor cancers. Hepatic peripheral tolerance mechanisms and hepatic monocyte-derived macrophages within the hepatic microenvironment have been proposed as agents of T cell-specific apoptosis and subsequent elimination of crucial antigen-specific T cells leading to systemic immunosuppression (84). Liver metastases were shown to induce systemic tumor-specific CD8^+^ T cell loss by siphoning activated antigen-specific CD8^+^ T cells from the circulation. Furthermore, the presence of liver metastases creates a hepatic microenvironment for apoptosis of activated antigen-specific Fas^+^CD8^+^ T cells following the interaction of these cells with tumor-educated and exposed FasL^+^CD11b^+^F4/80^+^ macrophage-derived hepatic myeloid cells. Single-cell RNA sequencing of cells within the hepatic microenvironment in mice models with liver tumors demonstrated a decreased proportion of cells within T cell clusters. Moreover, within the activated T cell population in the hepatic microenvironment, a more enriched population of apoptosis gene signatures was found in those mouse models with liver metastases compared to those without.

Patients with symptomatic CNS melanoma metastases or those requiring steroids following treatment of CNS metastases have previously also exhibited poor treatment outcomes. Patients with melanoma CNS disease historically had a median survival of about 4 months and are further limited by poor functional status, extracranial disease, and age (88, 89). Moreover, very little is known about predictive biomarkers and markers of response in the CNS. Dedicated studies are sorely needed to address this patient population. Ongoing studies, along with clinical experience, suggest that patients with melanoma brain metastases or those with symptomatic CNS lesions and requiring steroids may benefit from immunotherapy.

The phase II CheckMate 204 trial studied nivo + ipi in patients with untreated melanoma brain metastases (90). Patients were divided into two study cohorts: one asymptomatic with no neurologic symptoms or steroid use and the second cohort with neurologic symptoms or in need of steroid use. In the asymptomatic cohort, the intracranial clinical benefit rate (CBR), the proportion of patients with CR + PR + SD for ≥6 months, was 58.4%. In the symptomatic cohort, the intracranial objective response rate was only 16.7% and the CBR was 22.2%. While some intracranial antitumor activity was noted in the symptomatic melanoma brain metastatic group, studies to examine the biologic mechanisms to immunotherapy resistance in these hard-to-treat populations are needed. Similarly, the phase II ABC trial study showed similar results in patients treated with nivo + ipi combination compared with nivolumab monotherapy in patients with asymptomatic brain metastases with no previous local brain therapy (91, 92). A recent systematic review demonstrated a median OS of only 9.0 months in patients with melanoma brain metastases treated with immunotherapy (93).

Recent data presented at the European Society of Melanoma Congress 2021 have reported encouraging results in the management of melanoma brain metastases with the nivo + ipi combination. The 3-year study results of the CheckMate 204 study demonstrated that with a minimum follow-up of 34 months in patients with asymptomatic brain metastases, the investigator-assessed intracranial progression-free survival rate (icPFS) was 54% and the OS rate was 72%. In those patients with symptomatic brain metastases, 36-month icPFS was 19% and OS was 37% (94). Reassuringly, these results suggest that the combination of nivo + ipi serves as a viable standard of care for patients with brain metastases, both asymptomatic and symptomatic, providing hope of a treatment for this vulnerable population.

## Neoadjuvant ICB Studies and Biomarkers

Checkpoint blockade therapies have shown additional promise in the neoadjuvant setting. For an additional population of melanoma patients who may derive long-term benefits from ICB therapy, those patients with resectable clinical stage III melanoma may benefit from ICB treatment prior to surgical resection. Melanoma is particularly well suited for neoadjuvant approaches given the potential of improved surgical outcomes from surgery with control of micrometastatic disease prior to surgery and with the high propensity for regional disease that is safely assessable for longitudinal sample and analysis (95). Several studies have demonstrated that high rates of pathologic complete responses and impressive recurrence-free survival rates are attainable following neoadjuvant ICB treatment for stage III melanoma (96–100).

Neoadjuvant ICB studies also identified potential biomarkers of response in terms of early pathologic responses and IFNγ signatures. In a pooled analysis from the International Neoadjuvant Melanoma Consortium of six clinical trials of anti-PD-1-based ICB or BRAF/MEK-targeted therapy, here pathologic complete response (pCR) correlated with improved recurrence-free survival (2-year RFS with pCR 89% vs. no pCR 50%, *p* < 0.001) and OS (2-year pCR OS 95% vs. no pCR 83%, *p* = 0.027). Moreover, in patients with pCR, near pCR, or partial pathologic response, few relapses were seen (2-year RFS 96%), with no patient deaths from melanoma compared with 2-year RFS of patients with pCR from targeted therapy of 79%. Using pathologic response as an early surrogate endpoint for clinical trials may serve as an additional new benchmark for ICB treatment in melanoma (95).

In a separate study, the OpACIN trial compared neoadjuvant with adjuvant ICB nivo + ipi combination therapy. Adjuvant ICB with both ipilimumab and nivolumab had been shown to be associated with improved relapse-free survival, overall survival, and distant metastasis-free survival compared with placebo of stage III melanoma patients (101, 102). Here, patients with palpable stage III melanoma were randomized 1:1 to receive nivo + ipi whether as four courses in the adjuvant setting or as two courses prior to surgery and two post-surgical courses in the neoadjuvant arm. Pathologic responses were seen in 78% of patients treated in the neoadjuvant arm, where none of the patients in the arm relapsed with a median follow-up of 25.6 months. Additionally, this study reported that IFNγ signature may be used as a biomarker of response in patients treated with neoadjuvant nivo + ipi. Here, a high or intermediate IFNγ RNA signature was a predictor of clinical outcome of patients treated with neoadjuvant nivo + ipi, where none of the patients with a high IFNγ signature had relapsed. Low IFNγ signature was associated with relapse after nivo + ipi, independent of neoadjuvant or adjuvant treatments (97).

The use of favorable IFNγ signatures may therefore serve as a biomarker in additional neoadjuvant ICB trials. In another study of neoadjuvant/adjuvant anti-PD-1 therapy in stage III/IV melanoma, all patients who experienced a rapid antitumor response with a complete or major pathologic response after a single dose of anti-PD-1 remained disease free 3 weeks following treatment. Here, rapid clinical and pathologic responses were associated with the accumulation of exhausted CD8^+^ T cells 3 weeks following single-dose neoadjuvant/adjuvant anti-PD-1 treatment. A strong neoadjuvant response signature (NRS) was associated with genes involved in adaptive immune response, T cell activation, and migration that also correlated with post-treatment tumor-infiltrating lymphocyte (TIL) responses and RFS. An 18-gene IFNγ T cell-inflamed signature, GEP18, was associated with clinical response in this stage III anti-PD-1-treated melanoma setting. The NRS here strongly enriched for T effector or T memory CD8^+^ T cell transcriptional factors compared with naive CD8^+^ T cells. The importance of pre-existing exhausted T cell populations here was again evident with a stronger enrichment of the exhausted CD8^+^ T cell population compared with the effector T cell population within the neoadjuvant response signature (99). Moreover, a separate study of melanoma patients treated in the neoadjuvant setting demonstrated that treatment stratification based on exhausted T cell (T_ex_) frequency is possible and may limit adverse events associated with neoadjuvant nivo + ipi. The frequency of Tex cells was defined as the percentage of CD8^+^ T lymphocytes in pretreatment samples that expressed both inhibitory receptors PD-1 and CTLA-4 within the intratumoral CD8^+^ T cell population. Here, of the neoadjuvant-treated patients, 10 received anti-PD-1 and 7 nivo + ipi. Of the total patients, 12 achieved a CR, 4 a PR, and 1 with SD. Surgery was performed on 11 of the 17 patients with 8 attaining a pathologic CR. Median RFS and OS were not reached. In this study, patients who received neoadjuvant ICB were enriched for a high Tex population with a mean frequency of 25.7%, demonstrating that immune profile directed neoadjuvant therapy for locally advanced melanoma has the potential of high objective response rates (103).

Finally, early imaging at 3 weeks with ^18^F-fluorodeoxyglucose (FDG) positron emission tomography–computed tomography (PET-CT) scans at baseline and before surgical resection demonstrated that consistent with pathologic response, radiographic responses were observed after one dose of anti-PD-1, where decreases in tumor size with a ≥20% decrease in tumor size were seen in patients who remained tumor free. Conversely, here, FDG avidity was not associated with response (99). Separately, another phase II study of neoadjuvant nivo + ipi versus nivolumab monotherapy also demonstrated that the role of imaging could serve as an indicator of response in the neoadjuvant setting. Treatment with nivo + ipi yielded high ORR (73%) when measured by RECIST 1.1 as well as pathologic complete response rates (45%) but with substantial grade 3 treatment-related adverse events (73%) compared with modest responses with neoadjuvant nivolumab monotherapy (25% ORR, pCR 25%) though with lower toxicity (8% grade 3 treatment-related AEs) (98). Taken together, pathologic complete responses, IFNγ and exhausted T cell populations, and decreases in tumor size *via* imaging studies are strong forerunners to serve as robust biomarkers of neoadjuvant ICB response.

## Molecular Determinants of Tumor Response to ICB

With the diverse options for melanoma treatment with ICB alone or in combination, the push for predictive biomarkers to determine the ideal patient populations for each treatment type and to identify early, likely responders to treatment has been a topic of active study. Profiling of tumors of patients and tumor microenvironments for mutations and T cell-inflamed gene expressions is ongoing to help determine the optimal treatments for patients in the frontline or retreatment setting. For patients who have suboptimal responses to ICB, additional studies are ongoing to determine how to best boost the immune response. Additionally, given the poor reproducibility among currently available biomarkers, there remains a paucity of melanoma-specific predictive factors of ICB response that can functionally and reliably be used in the clinical setting.

## Tumor Biomarkers, PD-L1

Correlation with response was noted in tumor mutations, neoantigen load, and immune-related gene expression in tumor tissue with CD8^+^ T cell infiltrates. Activated tumor-infiltrating T cells have been shown to be markers of ICB response, yet the predictive value of these tests has yet to be fully studied (104). PD-L1 expression was an early front-runner as a predictive biomarker. Several early clinical trials explored PD-L1 as a surrogate of ICB response; though given a multitude of reagents and antibodies used across several assays, the reliability of PD-L1 as a biomarker of response remains variable, and PD-L1 expression status has varied in its prediction of melanoma response to ICB (105).

KEYNOTE-001 reported that PD-L1 expression in pretreatment tumor biopsies of melanoma correlated with response rate, PFS, and OS, though it was also observed that patients with PD-L1-negative tumors also exhibited treatment response (106). KEYNOTE-066 reported OS benefit with pembrolizumab in melanoma compared with ipi across all subgroups except for a small subgroup of patients with PD-L1-negative tumors (2). The phase II CheckMate 064 trial examined patients who received ipi for 12 weeks then nivo for 12 weeks with subsequent nivo maintenance compared with patients who received nivolumab prior to ipilimumab. A higher proportion of patients with baseline PD-L1 expression of 5% or more achieved a response in both sequential treatment groups compared with those with <5% PD-L1 expression. Yet, a higher proportion of patients in the nivo then ipi group were evaluable for baseline PD-L1 expression and had PD-L1 of 5% or more compared with patients in the ipi then nivo group (30).

CheckMate 066 included patients treated with nivolumab versus dacarbazine and showed that nivolumab improves OS in previously untreated melanoma patients and showed that given the magnitude of clinical benefit observed in patients who got nivolumab, PD-L1 status alone is not helpful in the selection of patients for nivo treatment (26). ECHO-301/KEYNOTE-252 stratified patients by PD-L1 expression and BRAF V600 mutation status and randomly assigned 1:1 to the IDO-1 inhibitor plus pembrolizumab or placebo plus pembrolizumab (107).

KEYNOTE-028 examined the T cell-inflamed gene expression profile, PD-L1 expression, and tumor mutational burden efficacy in patients treated with pembrolizumab across 20 solid tumor cancers. Patients with PD-L1-positive tumors were treated with pembrolizumab for 2 years or until confirmed disease progression or toxicity prompted treatment discontinuation. Higher response rates and longer PFS were seen in tumors with higher T cell-inflamed gene expression profiles, PD-L1 expression, and/or tumor mutation burden (TMB). Correlations of TMB with T cell gene expression profile and PD-L1 were low. Patients with high TMB and inflammatory markers (T cell gene expression profile or PD-L1) were the patients with the highest likelihood of response (108).

Despite the studies utilizing PD-1 as surrogates and potential markers of response, the following question remains: why do patients with PD-L1-negative tumors respond and why do the subset of patients with PD-L1-positive tumors not respond to PD-1 pathway blockade? Besides PD-L1 tumor cell expression, PD-L1 expression on immune cell-infiltrating tumors has become another avenue of exploration as a potential predictor of clinical response (109). Additionally, PD-L1 testing based on mRNA level has been feasible, though correlation between PD-L1 expression *via* IHC and RT-PCR is variable between the types of antibody being used. No difference was found between PD-L1 expression between responders and non-responders to therapy with ipilimumab (110).

## Tumor Microenvironment, TILs

The TME and the composition of cells within the TME have been identified as potential predictive markers of response in melanoma (111, 112). The current hypothesis proposes that anti-CTLA-4 ipilimumab leads to a more favorable tumor environment for improved efficacy with concurrent or sequential anti-PD-1 therapy. Ipilimumab is thought to increase TILs and IFNγ inducible genes in the TME (113, 114). In turn, this increase in cell populations in the TME increases PD-L1 expression (115). With the increase in PD-L1 expression, the primary ligand for PD-1, the hypothesis proposes an increase in the proportion of patients who experience an improved objective response as well as overall survival in those treated with PD-1.

Classification of the TME into different subtypes has emerged as a way to classify response to ICB based on the presence or absence of TILs and PD-L1 status. The most immunogenic tumors are those with pre-existing TIL^+^/PD-L1^+^ in the TME and are thought to be the most likely to respond to ICB. Those with TIL^−^/PD-L1^−^ tumors are the least likely to respond to ICB and seen as an immunologic desert. The TIL^−^/PD-L1^+^ tumors are thought to be “immune excluded” with a functional PD-L1 pathway and may most benefit from combination ICB to optimize lymphocyte recruitment to the tumor bed. Finally, TIL^+^/PD-L1^−^ tumors are thought to need alternative strategies beyond the conventional ICB CTLA-4/PD-1 therapies to target additional immunosuppressive pathways (116, 117).

Adaptive immune resistance *via* CD8^+^ T cells upregulating PD-L1 on melanoma tumor cells has been observed at the invasive tumor margin. *Via* histopathology, of the tumor tissue samples obtained before and after anti-PD-1 treatment, increased expression of CD8^+^ PD-1 or PD-L1 at the invasive tumor margin correlated with response to anti-PD-1 treatment. Together with a more clonal TCR repertoire, this model suggested a predictive model based on pre-existing CD8^+^ T cell expression at the tumor-invasive margin following anti-PD-1 treatment may be indicative of response (109).

The gene expression profile of the TME has also been examined as potential predictive biomarkers of ICB response (118). The concept of a T cell-inflamed TME has emerged predictive factors of response to ICB, vaccines, and IL-2 (20, 113, 119). This inflamed TME has been observed in the setting of tumor-infiltrating CD8^+^ T cells secreting IFNγ, triggering an intratumoral antitumor inflammatory state (120). Thus, across several cancer subtypes with immunotherapy treatment, the inflamed TME *via* the increase in IFNγ-associated gene expression scores is predictive of response to anti-PD-1 therapies (namely, pembrolizumab). Furthermore, the lack of IFNγ-associated gene expression has strongly correlated to a lack of ICB treatment benefit (121–124).

Other markers of response explored have been TMB and T cell-inflamed gene expression profiles (GEP). Both have shown joint predicative utility in stratifying responders and non-responders to pembrolizumab and may be capturing distinct features of neoantigenicity and T cell activation. A study evaluated samples from four KEYNOTE trials and examined the joint predictive utility of the TMB and T cell-inflamed GEP to identify responders versus non-responders to pembrolizumab. In melanoma, both TMB and GEP scores were positively associated with BOR, with an area under the receiver operating characteristic curve (AOROC) value of 0.602. The correlation between TMB and GEP with predicting response was low (Spearman correlation coefficient *r* = 0.252, *p* < 0.05). TMB showed no association with PD-L1 in melanoma (MEL score *r* = 0.049, *p* = 0.65), whereas GEP was more significantly correlated with PD-L1 (*r* = 0.53, *p* < 0.0001). The most pronounced PFS-associated hazard ratios were observed for TMB high GEP high tumors. In melanoma, the percentage of UV-light-induced mutations correlated with TMB (*r* = 0.77; *p* < 1 × 10^−10^) and was significantly associated with response (*p* = 0.02). This suggests that non-synonymous mutations arising from a variety of mutagenic processes are capable of enhancing the antigenicity of tumors with comparable effects on the response to anti-PD-1 treatment (125).

## Durability of ICB Response: Understanding the Unique Immune Cell Populations

Immunologic memory is thought to be a characteristic of durable responses to ICB therapy. CTLA-4 inhibition increases T cell priming and promotes T cell diversity, acting on both functionally impaired cytotoxic T cells and helper T cells, while PD-1 inhibition promotes the clonal expansion of previously activated, functionally impaired CD8^+^ cytotoxic T cells (33, 126–128). ICB therapies have been shown to act on different populations of immune cells at different stages of immune activation, with the potential of a select immunologic memory T cell subset contributing to durable responses to ICB therapy.

The proportion of pre-existing CD8^+^ T cells at the invasive tumor margin has been shown to correlate with increased clinical response to anti-PD-1 treatments (109, 129). Intratumoral PD-L1 expression is induced by the IFNγ signaling pathway, chromosomal alterations, or a constitutive oncogenic signaling pathway (130). The IFNγ signaling pathway is thought to be the mechanism behind adaptive resistance, a defense mechanism of tumor cells against the immune system attack by IFNγ secreting CTLs and T_h_1 cells. Consequently, a link between clinical efficiency and PD-L1 expression, CD8^+^ T cell tumor infiltration and somatic burden, or the number of neo-antigens originating from increased mutated genes and abnormal proteins has been proposed (131).

CD4^+^ T cells have been shown to promote tumor regression *via* IL-2 secretion, by directly eliminating cancer cells or by augmenting tumor-specific CD8^+^ T cell function (132–135). The role of CD4^+^ T cells in ICB continues to be an active area of exploration as markers of long-term survival in melanoma, though not all studies have made distinctions between regulatory and effector CD4^+^ cells. A study examined pre- and post-treatment peripheral blood samples from patients with malignant melanoma treated with anti-PD-1 monoclonal antibodies. Using mass cytometry assays and screening by high dimensional clustering, three microclusters of CD4^+^ T cells and a subset of central memory CD4^+^ T cells with a CD27^+^FAS^−^CD45RA^−^CCR7^+^ phenotype were identified in long-term survivors to anti-PD-1 and not identified in non-responders to anti-PD-1 therapy (136). CD27 is a lymphocyte-specific member of the TNF receptor superfamily, expressed by CD45RA^+^CCR7^+^ naive CD4^+^ T cells, and is further upregulated following T cell receptor signaling, yet decreased expression with effector CD4^+^ T cell differentiation (137, 138). FAS is a member of the TNF receptor superfamily and has been shown to have pro- and anti-apoptotic T cell effects (139). Activated T cells have been known to express FAS, while naive CD4^+^ T cells do not. With the expression of CD27^+^ and FAS^−^ T central memory CD4^+^ T cells, this intermediate population may be indicative of a fraction of cells differentiating from naive to central memory cells. This intermediate population may be indicative of cells just egressed from draining lymph nodes to peripheral blood following TCR stimulation *via* cognate antigens. However, CD27^+^FAS^−^ central memory CD4^+^ T cells have not been shown to express PD-1, suggesting that therapeutic anti-PD-1 monoclonal antibodies do not directly interact with this T cell subset.

Transcriptome and immune profiling of melanoma tumor biopsies of patients treated with anti-PD-1 monotherapy or nivo + ipi have identified activated T cell signatures and populations of T cells unique to the responders to ICB. Transcription factors TBET and eomesodermin (EOMES) are drivers of immune cell development and have been shown to link the long-term renewal of memory CD8^+^ T cells to their effector potency. TBET and EOMES are master regulators of effector T cell and memory formation and induce helper T cell effector function in CD8^+^ cytotoxic T cells *via* the upregulation of IFNγ and granzyme B (GZMB), a cytotoxic granule and T cell activation marker (140, 141). Taken together, transcriptome and immune profiling have identified a population of CD8^+^/CD4^+^EOMES^+^CD69^+^CD45RO^+^ (and TBET^high^) effector memory T cells in responders to nivo + ipi. This population of cells though has not been seen in non-responders to the combination therapy. Additionally, this effector memory T cell population was associated with longer PFS and tumor shrinkage in anti-PD-1 monotherapy-treated patients (142). This specific memory T cell population associated with the response to anti-PD-1 monotherapy and nivo + ipi combination therapy demonstrates the potential of utilizing immune infiltrates as markers of durable response to ICB.

Additionally, a subset of immune effector cells has been identified as a way to identify patients who are likely to respond to ICB treatment. This population of peripheral T cells with the CD3^+^/CD4^−^/CD8^+^/CD45RA^−^/CD45RO^high^/CD27^−^/CCR7^−^ signature following one cycle of ICB has been associated with T cell evolution in response to treatment. This dynamic awakening of the immune system was identified using T cell receptor sequencing in plasma cell-free DNA and peripheral blood mononuclear cells. Along with a phenotypic analysis of peripheral T cell subsets of melanoma patients treated with ICB, early peripheral T cell turnover and TCR repertoire dynamics are associated with ICB response. Additionally, the timeline of this immune awakening within 3 weeks of ICB initiation provides promise to monitor patient responses using minimally invasive liquid biopsies (143).

Checkpoint blockade has also been shown to mediate the response of tumor-infiltrating CD8^+^ T lymphocytes. The chronic activation of this TIL population has been thought to create a state of terminal differentiation or exhaustion of these tumor-specific T cells. Given this, the identification of a subset of exhausted T cells and central memory cells associated with the expression of PD-1 and transcription factor Tcf1 has been studied. A population of Tcf1^+^PD-1^+^ TILs has been shown to mediate the response to ICB and, in turn, generate additional populations of Tcf1^+^PD-1^+^ and differentiated Tcf1^−^PD-1^+^ cells. The Tcf1 transcription factor was not required for the generation of the Tcf1^+^PD-1^+^ TIL population, though essential for the stem-like function of these cells. Additionally, the ablation of this Tcf1^+^PD-1^+^ TIL population has been associated with restricted responses to ICB. Taken together, this study proposes that checkpoint inhibition relies less on the reversal of T cell exhaustion and more on the proliferation of this stem-like TIL subset, which is likely implicated in ICB response (144).

Differences between biomarkers associated with efficacy to CTLA-4 versus PD-1 monoclonal antibody treatments have also emerged. A study utilizing mass cytometry profiling of peripheral blood mononuclear cell (PBMC) samples from melanoma patients suggested a difference in anti-PD-1-treated but not anti-CTLA-4-treated patients. In those treated with anti-PD-1, differences between responders and non-responders with a CD69 and MIP-1ß NK cell population were seen. Here, natural killer cell subsets, but not memory CD4^+^ or CD8^+^ T cell subsets, correlated with clinical response to anti-PD-1 therapy, whereas these CD4^+^ or CD8^+^ memory T cells differed between responders and non-responders to anti-CTLA-4 therapy (145).

Melanoma bulk-tumor transcriptomic and single-cell (sc)RNAseq data have identified other potential biomarkers of response and survival in patients treated with sequential ICB therapy (anti-CTLA-4 then anti-PD-1). In patients treated with sequential anti-CTLA-4 then anti-PD-1, the CD8^+^/CD4^+^ T cell signature associated with IFNγ signaling or cytolytic activity failed to predict an antitumor response. Conversely, early memory CD8^+^/CD4^+^ T cell signatures [associated with the transcription factor, T cell factor 1 (TCF-1)-driven stem-like transcriptional program, characteristic of resisting cell death or apoptosis] have been shown to be predictors of ORR to ICB and survival following anti-CTLA-4 and anti-PD-1 sequential therapy. This suggests that sequencing of ICB therapy may impact the T cell repertoire and influence the value of predictive immune biomarkers (146).

Separately, chemotherapy and immunotherapy treatment scheduling may also affect the ICB response. Preclinical mouse models have demonstrated that tumor draining lymph nodes affect the tumor antigen-specific T cell response. Removal of tumor draining lymph nodes concurrently with established primary tumors did not affect the ICB response on localized secondary tumors given the distribution of antigen-specific T cells in peripheral lymphatic organs and the immunotolerance in tumor draining lymph nodes. Yet, in this study, tumor responses were proven with the sequential administration of 5-fluorouracil (5-FU) and ICB compared with the concurrent administration of 5-FU and ICB where immune profiling revealed that the utilization of 5-FU as an induction treatment decreased immunosuppressive cells in the tumor microenvironment, increased tumor visibility to immune cells, and limited chemotherapy-induced T cell depletion. Here, in preclinical models, traditional cytotoxic treatment in sequence with ICB influenced immunotherapy response in localized secondary tumors and may be a strategy utilized in the clinical setting to induce long-term tumor responses (147).

**Figure 1:**
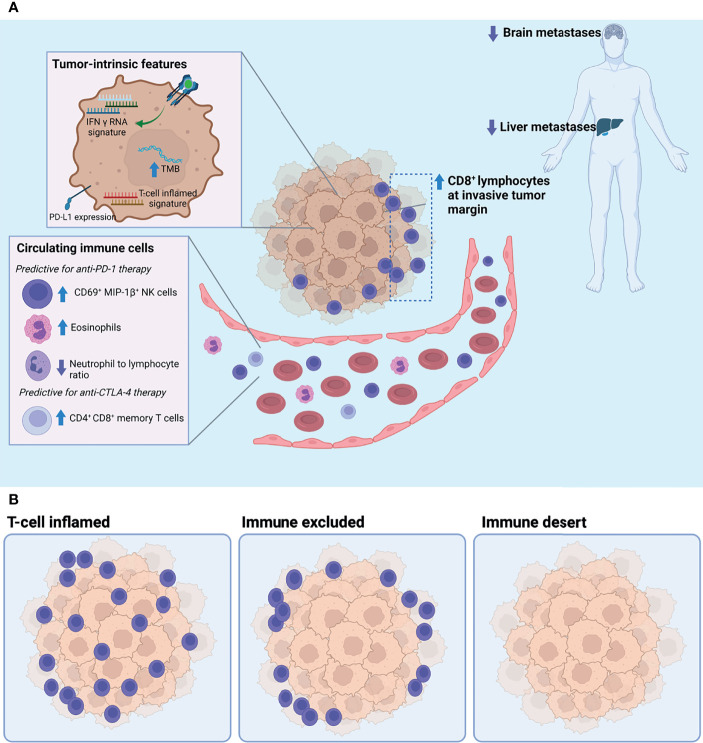
**(A)** Tumor-intrinsic and circulating biomarkers associated with response to immune checkpoint blockade. **(B)** Three archetypical tumor microenvironments defined by the degree of T cell infiltration: T cell inflamed, immune excluded, and immune desert. Of these, the T cell-inflamed phenotype has been positively associated with response to immune checkpoint blockade. TMB, tumor mutational burden.

Given these promising hints of potential biomarkers, the importance of understanding the biology surrounding the tumor microenvironment, the modulation of NK cells, and the role that both effector or exhausted CD8^+^ T cells and TILs play in responders versus non-responders to ICB therapy is imperative to develop clinically informative predictive biomarkers of response (**Figure 1**).

## Conclusion

Across several clinical trials, the long-term survivorship of patients treated with immune checkpoint blockade therapies continues to shed light on the durability and promising nature of the treatment of those with advanced melanoma. Several trials with follow-up and landmark OS rates of at least 5 years demonstrate just how widely immunotherapies have revolutionized the landscape of melanoma treatment in the last decade. The potential for long-term survival, durable responses, and even possible cure models in melanoma provides an abundance of hope. For patients who do not experience the benefit of long-term response, escalation of care with either rechallenge of ICB or additional therapies is under study and development.

With long-term survival now more attainable than ever, clinicians are looking toward markers that may stratify melanoma patients for ICB therapies with durable clinical responses. Research currently focused on identifying both robust prognostic and predictive biomarkers of response to ICB is underway. To maximize therapeutic potential and minimize undesirable toxicities, the translational potential of neoadjuvant pathologic complete responses, baseline blood chemistry serologies, tumor and microenvironment TIL composition, and additional immune cell populations contributing to lasting T cell memories have been identified as potential biomarkers of long-term response. The future development of therapies alone or in combination with current ICBs and improvements in the diagnostic accuracy of biomarkers are promising to achieve long-term survival and possible cure for advanced melanoma. 

## Author Contributions

KL wrote, reviewed, and edited the article. AW and MP reviewed and edited the article. JS designed the figure. All authors contributed to the article and approved the submitted version.

## Funding

The authors were supported by the Cancer Center Support Grant P30 CA08748 from the National Institutes of Health/National Cancer Institute.

## Conflict of Interest

The authors declare that the research was conducted in the absence of any commercial or financial relationships that could be construed as a potential conflict of interest.

## Publisher’s Note

All claims expressed in this article are solely those of the authors and do not necessarily represent those of their affiliated organizations, or those of the publisher, the editors and the reviewers. Any product that may be evaluated in this article, or claim that may be made by its manufacturer, is not guaranteed or endorsed by the publisher.
